# Preparation and Characterization of a New Monoclonal Antibody Specific Against *Lawsonia intracellularis* and Its Application in Indirect Immunofluorescence and Immunocytochemistry Assay

**DOI:** 10.3389/fvets.2021.753610

**Published:** 2021-11-29

**Authors:** Ning Xiao, Jiannan Li, Minxue Li, Yuting Hu, Huixing Lin, Hongjie Fan

**Affiliations:** ^1^MOE Joint International Research Laboratory of Animal Health and Food Safety, College of Veterinary Medicine, Nanjing Agricultural University, Nanjing, China; ^2^Jiangsu Co-innovation Center for the Prevention and Control of Important Animal Infectious Diseases and Zoonoses, Yangzhou University, Yangzhou, China

**Keywords:** proliferative enteropathy, *Lawsonia intracellularis*, Omp2 protein, monoclonal antibody, indirect immunofluorescence assay, immunocytochemistry

## Abstract

Proliferative enteropathy (PE) is an infectious enteric disease caused by *Lawsonia intracellularis* (*L. intracellularis*) and is endemic in pig herds worldwide. However, a *L. intracellularis*-specific monoclonal antibody plays an important role in the evaluation of *L. intracellularis* infection *in vitro*. Therefore, the objective of this study was to produce and identify the characteristics of a new monoclonal antibody against the outer membrane protein (Omp2) of *L. intracellularis* and apply it in an indirect immunofluorescence assay (IFA) and immunocytochemistry (IHC). The results indicated that three highly specific monoclonal antibodies against the Omp2 protein (4D9, 3G2, and 7G5) of *L. intracellularis* were obtained by using purified Omp2 as an immunogen, the titers of ascitic fluids of 4D9, 3G2, and 7G5 cells were 1:2,048,000, 1:512,000, and 1:256,000, respectively. IFA analysis showed that the 4D9, 3G2, and 7G5 have no cross-reactivity with other enteric bacteria commonly found in the ilea of pigs or closely related to *L. intracellularis*, such as *Desulfovibrio, Bilophila wadsworthia* (*B. wadsworthia*), *Salmonella choleraesuis* (*S. choleraesuis*), *Salmonella typhimurium* (*S. typhimurium*), *Escherichia coli* (*E. coli*), and *Brachyspira hyodysenteriae* (*B. hyodysenteriae*). IFA and IHC results indicated that the monoclonal antibodies can be successfully used as primary antibodies to detect *L. intracellularis* in infected cells and in the crypt of the ileum from infected tissues of PE. Our findings suggested that the new monoclonal antibody specific against *L. intracellularis* will be useful for the evaluation of *L. intracellularis* infection *in vivo* and *in vitro*.

## Introduction

Proliferative enteropathy (PE) is an infectious enteric disease characterized by thickening of the ileal wall as a result of enterocyte proliferation associated with the presence of *Lawsonia intracellularis* (1, 2). *L. intracellularis* causes PE in various species, such as pigs, horses, hamsters, dogs, and non-human primates (3–6). Porcine intestinal adenomatosis (PIA) is considered to be the chronic form of PE, which leads to mild diarrhea and reduced growth in young pigs aged 8–20 weeks (7, 8). Proliferative hemorrhagic enteropathy (PHE), an acute form of PE, often occurs in older finisher pigs, gilts, and sows, is characterized by bloody diarrhea, and often leads to sudden death (7, 8).

*L. intracellularis* has been widely spread in more than 20 countries, such as China, Canada, Brazil, Finland, France, South Africa, Greece, and India (9, 10), and the disease causes more than $1.63 per infected pig due to bloody diarrhea, sudden death, decreased body weight, stunted growth, and increased costs of feeding and medication worldwide (11–13). In Denmark, the herd prevalence detected by qPCR and ELISA were 90.0 and 91.7%, while in the United Kingdom, the herd prevalence detected by the commercial kit SVANOVIR® *L. intracellularis/Ileitis-Ab* was 100.0% (14). In the field of China, the seroprevalence of *L. intracellularis* antibodies in pigs on intensive farms was evaluated by a commercial blocking ELISA kit in 2014 (15), and the overall true prevalence of *L. intracellular*is animal seropositivity was 77% (95% CI, 70–83%). A higher rate of seroprevalence was found in fattening pigs, sows, and boars than in preweaning piglets and weaners (15). The results indicate that the infection of *L. intracellularis* is common in pig herds in China. Therefore, to control the infection and spread of PPE effectively, it is crucial to obtain the *L. intracellularis* strain isolated from China and prepare an effective PPE vaccine, while the prerequisite for successful isolation of *L. intracellular* is to prepare the specific monoclonal antibody to monitor the bacteria in tissue sample and infected cell monolayer.

Only a few laboratories around the world are capable of isolation and cultivation of *L. intracellularis* due to the fastidious microaerophilic obligate intracellular nature of this organism (6, 16, 17). To date, there is no information about successful isolation and maintenance of *L. intracellularis* infection *in vitro* in China. This is mainly because the cells are easily contaminated after being inoculated with the intestinal homogenate of infected pigs as *L. intracellularis* resides in a contaminated environment. Furthermore, sensitive and accurate methods suitable for determination of the viability and detection of *L. intracellularis* directly from the cell culture are scarce. Although a variety of methods are available for evaluation of a highly *L. intracellularis*-infected cell monolayer when isolating and cultivating *L. intracellularis* from clinical intestinal samples, such as PCR (18), fluorescent *in situ* hybridization (FISH) (19), and immunoperoxidase monolayer assay (IPMA) (20), these methods also have some limitations. A major limitation of PCR is that it has no ability to differentiate between live and dead bacteria; Furthermore, PCR results only indicate the nucleic acid of infected cells and unable to confirm whether the bacteria has entered the intestinal epithelial cells. Although FISH is well-standardized and easily performed, it requires experience in interpreting the results. A previous study has shown that autofluorescence could produce false-positive results, and the use of PNA probes remains expensive, and the sensitivity of FISH remains lower than PCR in the case of analyses from primary materials (21, 22). Moreover, the infected cells also could be detected by IPMA after being incubated for 5 days in a gas concentration of 8.0% oxygen (O_2_), 8.8% carbon dioxide (CO_2_), and 83.2% nitrogen (N_2_), which is time consuming (23). Therefore, the PCR, FISH, and IPMA methods are not the most effective way to monitor the infected cells when isolating *L. intracellularis*. However, immunofluorescence assay (IFA) presents many advantages such as repeatable, sensitive, and less time consuming (24). Furthermore, the infected cells could be precisely detected after being incubated for 3 h in a gas concentration of 8.0% O_2_, 8.8% CO_2_, and 83.2% N_2_, which will improve the detection efficiency when isolating *L. intracellularis* from an infected intestine.

Therefore, the objectives of the present study was to produce and identify the characteristics of a new monoclonal antibody against outer membrane protein (Omp2) of *L. intracellularis* and apply it in an indirect immunofluorescence assay (IFA) and immunocytochemistry (IHC). Our findings may lay the basis for evaluating the monolayer of McCoy, IEC-18, IPEC-J2, and many other cell lines and tissues infected with *L. intracellularis*.

## Materials and Methods

### Bacterial Strains, Plasmids, and Cell Lines

The *L. intracellularis* strain (a commercial live attenuated *L. intracellularis* vaccine, Enterisol®ileitis) was purchased from Boehringer Ingelheim Vetmedica, Germany, and *Escherichia coli* (CAU0751), *Salmonella choleraesuis* (CVCC2139), and *Salmonella typhimurium* (CVCC2220) were purchased from the China Veterinary Culture Collection Center (CVCC). *Desulfovibrio* (ATCC 27774) and *Bilophila wadsworthia* (ATCC 49260) were purchased from the American Type Culture Collection (ATCC). *Brachyspira hyodysenteriae* is courtesy of the Shanghai Animal Disease Prevention and Control Center. The pGex-6p-1 (+) vector was purchased from Novagen, USA. The myeloma cell line SP2/0 (stored in our laboratory) and rat small intestine cells (IEC-18; ATCC CRL 1589) were cultured in DMEM/high glucose (Gibco, USA) in a humidified 5% CO_2_ atmosphere at 37°C. All the cell culture media were supplemented with 10% fetal bovine serum (FBS, Gibco, USA). *L. intracellularis* was grown as described (5). In brief, 30% confluent IEC-18 monolayers were infected with *L. intracellularis* and incubated in an atmosphere of 83.2% nitrogen (N_2_), 8.8% carbon dioxide (CO_2_), and 8% oxygen (O_2_) for 7 days at 37°C.

### Expression, Purification, and Identification of the Outer Membrane Protein

The outer membrane protein (*Omp2*) gene sequences of *L. intracellularis* were selected and analyzed. The partial *Omp2* sequence GXNN strain was synthesized according to the 103–993 bp regions from *L. intracellularis* (no. EU621796.1) by Sangon Biotech Co., Ltd. (Shanghai, China) using restriction enzymes (*BamH* I and *Sal* I). The synthetic *Omp2* gene was cloned into the prokaryotic expression vector pGex-6p-1 (+). The positive recombinant plasmids were identified by enzyme digestion and sequencing and then transformed into *E. coli* Rosetta (DE3) competent cells. The expression of the recombinant Omp2 protein was induced with 0.3 mmol/L isopropyl-β-D-thiogalactopyranoside (IPTG, Sigma, USA) at 16°C for 18 h, and the protein was examined by SDS-PAGE. The recombinant protein was also detected by SDS-PAGE after purification with a high-affinity GSTRAP HP column (GE Healthcare Life Sciences, USA) according to the instructions of the manufacturer, and the concentration was determined with a NANODROP 2000 Spectrophotometer (Thermo, USA). In addition, Western blotting was performed to evaluate the reactivity of the purified Omp2 protein. The same method was described in the reference with some modifications (25). Briefly, the purified Omp2 protein was transferred onto a polyvinylidene fluoride (PVDF) membrane (Millipore Corporation, USA) after being separated by 12% SDS-PAGE. Then the membrane was blocked with 5% skim milk in TBST (20 mM Tris-HCl, 150 mM NaCl, 0.01% Tween-20, pH 7.4) overnight at 4°C, followed by incubation with an anti-*L. intracellularis*-positive antibody (SVANOVIR® *L. intracellularis/Ileitis-Ab*, Germany) for 1 h at 37°C. After three washes with TBST, the membrane was incubated with goat anti-pig IgG H&L (HRP) secondary antibody for 1 h at 37°C (Zhuangmeng, China). After three washes with TBST, the target protein bands were detected using 3,3-diaminobenzidine tetrahydrochloride (DAB), and the reaction was stopped by rinsing with distilled water and drying the membrane.

### Production and Identification of Monoclonal Antibodies Against the Outer Membrane Protein

The animal protocol used was approved by the Ethical Committee of the Faculty of Veterinary Science of Nanjing Agricultural University (approval number: IACUC20181009); and all experiments were performed in accordance with the relevant guidelines and regulations. The purified Omp2 protein was used to immunize 6-week-old BALB/c mice (Yangzhou University Comparative Medical Center, China). The mice were immunized with Freund's adjuvant (Sigma, USA) three times. Three days after the final immunization, the mice with the highest antibody titers were euthanized, and the spleen cells were harvested. Subsequently, splenocytes from immunized mice were fused with sp2/0 murine myeloma cells by using 50% polyethylene glycol (PEG 4000, Sigma, USA) according to a previously described method (26). The fusion cells were separated into 96-well-plates and cultured selectively in DMEM/high glucose containing 10% fetal bovine serum, 100 mM hypoxanthine, 400 mM aminopterin, and 16 mM thymidine. On day 5, 50 μl of HAT medium was added to each well of the 96-cell plates. On day 12, the HAT medium was completely replaced with fresh HT medium. After HAT/HT medium screening, the culture supernatants were analyzed by indirect enzyme-linked immunosorbent assay (ELISA). The positive hybridoma cells were repeatedly subcloned by the limiting dilution method until monoclonal hybridoma cells were obtained. Positive hybridoma cells were cultured in the abdominal cavity of paraffin-primed BALB/c mice to obtain ascitic fluid. The characteristics of the monoclonal antibodies (mAbs) were identified using a Pierce Rapid Isotyping Kit (Thermo Scientific, USA) according to the instructions of the manufacturer.

### Enzyme-Linked Immunosorbent Assay

The antibody titers of ascitic fluid were further determined by ELISA, as previously described (27). Briefly, the purified recombinant Omp2 protein was used as a coating antigen, the ascitic fluids were diluted in serial 2-fold dilutions from 1:1,000 to 1:2,048,000 and added to the wells in triplicate, and the negative control (unimmunized normal mouse serum) was included in each plate. After washing five times with PBST (0.5% Tween-20 in PBS), the plate was incubated with HRP-conjugated goat anti-mouse IgG (Abmart, China) at a ratio of 1:5,000 for 1 h at 37°C. The color was developed with 3,3′,5,5′- tetramethylbenzidine (TMB), and the reaction was stopped by 2 M H_2_SO_4_. The OD_450nm_ was measured in a micro titer plate reader (Bio-Rad Benchmark Plus). The cutoff value was determined when the OD_450nm_ ratio (OD_450nm_ of purified antibody/OD_450nm_ of unimmunized rabbit serum) was above 2.1. The antibody titer was defined as the highest dilution that yielded a net OD_450nm_ value greater than the calculated cutoff.

### Western Blot Analysis

The expressed Omp2 proteins were subjected to gel electrophoresis on 12% SDS-PAGE after denaturation with 2 × SDS loading buffer at 100°C for 3~5 min. The protein bands were transferred to PVDF membranes. The membranes were blocked with 5% skim milk in TBST overnight at 4°C. After washing three times in TBST, the membranes were incubated with mAbs against Omp2 protein, positive control (mouse serum against Omp2 protein, collected from the last injection) or negative control (unimmunized normal mouse serum) at 37°C for 1 h. After washing with TBST, the membranes were immersed in goat anti-mouse HRP-conjugated polyclonal serum at 37°C for 1 h. After washing in the same way, the membranes were immersed in DAB, and the reaction was stopped by rinsing with distilled water and drying the membrane.

### Assessment of Specificity of Monoclonal Antibodies

The cross-reactivity of the mAbs was detected by IFA. Briefly, about 1 × 10^5^ *L. intracellularis*, 1 × 10^5^ CFU of *Desulfovibrio, B. wadsworthia, S. choleraesuis, S. typhimurium, E. coli*, and *B. hyodysenteriae* were tested using a bacterial suspension to infect IEC-18 cells. The cell cultures were detected after being incubated at 37°C in 8.0% O_2_, 8.8% CO_2_, and 83.2% N_2_ in a humidified incubator for 3 h, the rest of the experiment steps were the same as before.

### Immunofluorescence and Immunohistochemistry Assay

Immunofluorescence assays were performed using IEC-18 cells seeded on 13-mm sterilized coverslips and cultured overnight to 30% confluence. The cells were infected with 1 × 10^5^ *L. intracellularis* at a multiplicity of infection (MOI) of 100 for 3 h and 7 days in a tri-gas incubator with 83.2% N_2_, 8.8% CO_2_, and 8% O_2_ at 37°C (Gene Science AG300 plus, USA) according to methods described in the literature (28). Uninfected cells were used as negative control. The cells were washed with PBS three times and fixed with ice-cold 4% paraformaldehyde (−20°C) for 15 min, and the cells were permeabilized with 0.3% TritonX-100 for 10 min at room temperature. After blocking for 2 h at 37°C in 5% skim milk in PBS, the fixed cells were then incubated with a 1:200 dilution of mAbs for 1 h at 37°C. Unimmunized normal mouse serum and mouse serum against the Omp2 protein were used as negative and positive controls, respectively. The cell monolayers were rehydrated by rinsing three times with PBST. FITC-labeled goat anti-mouse IgG and goat anti-mouse IgG Alexa Fluor 594 were used as a secondary antibody, respectively, at a 1:1,000 dilution and incubated in the dark for 1 h at 37°C. After three washes with PBST, 200 μl of DAPI (4,6-diamidino-2-phenylindole dihydrochloride; Ziyi Reagent Co., Shanghai, China) staining solution was added to each well, and the nuclei were stained in the dark for 10 min at room temperature. After washing, the coverslips were removed and immediately examined by confocal laser scanning microscope (Nikon ECLIPSE Ti, Japan).

The monoclonal antibody was also evaluated by immunofluorescence (IF) staining and immunocytochemistry (IHC) in histological fragments of lesions suggestive of PPE. Briefly, infected (subclinical form of PE) and normal intestinal samples were obtained from commercial pigs in a slaughterhouse in Jiangsu Province, China. Subsequently, the samples of ileum were fixed by immersion in 10% neutral-buffered formalin. *L*. *intracellularis-*specific Omp2 monoclonal antibody (4D9, 3G2, and 7G5) was used to perform IF and IHC to stain *L*. *intracellularis* bacteria according to the previously reported methods (29, 30). Uninfected sample was used as negative controls at the same time.

## Results

### Expression and Purification of the Outer Membrane Protein

**Figure 1:**
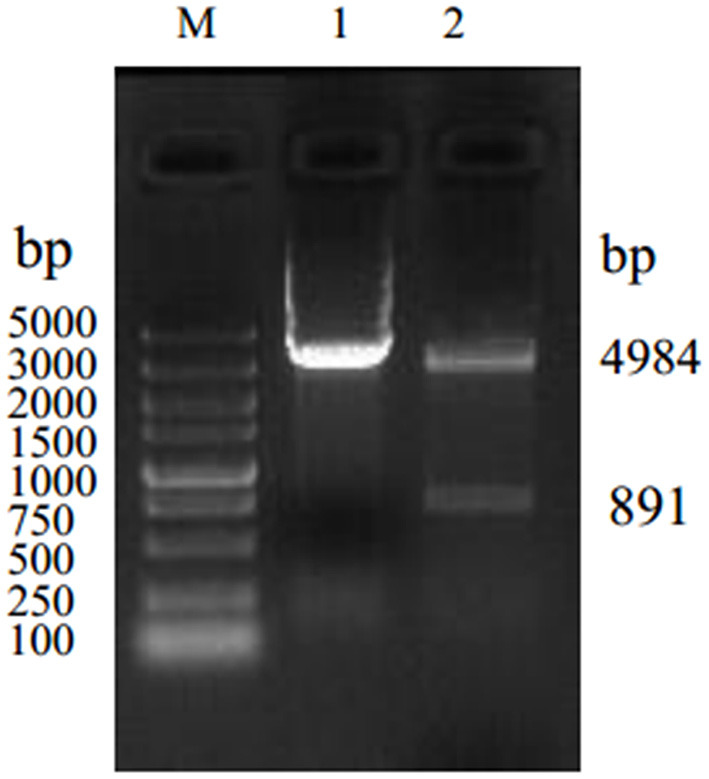
Identification of the recombinant plasmid pGex-6p-1-*Omp2* by restriction enzyme digestion with *BamH* I and *Sal* I. Lane M. DNA marker 5,000; lane 1: product of plasmid pGex-6p-1; lane 2: recombinant plasmid pGex-6p-1-*Omp2*.

**Figure 2:**
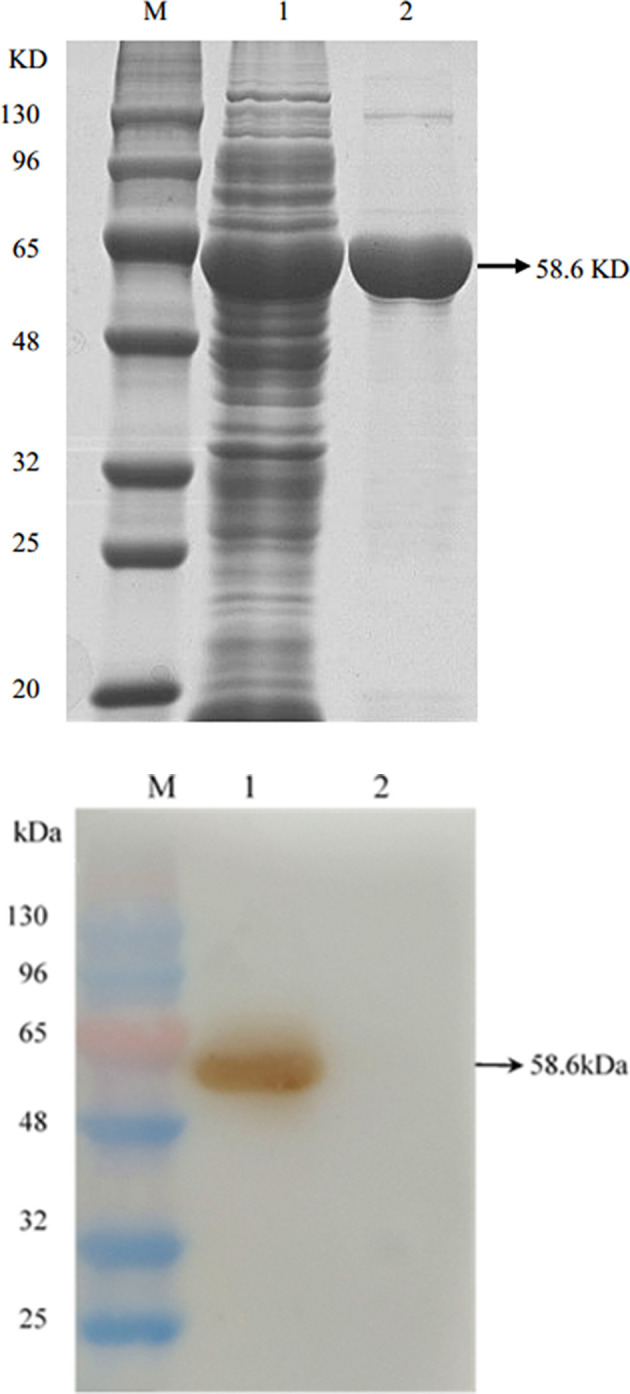
The expression and identification of Omp2 protein. **(A)** SDS-PAGE analysis of Omp2 expressed in *E. coli* BL21. Lane M: protein molecular weight standard; lane 1: soluble fraction of the induced bacterial lysate, lane 2: purified Omp2 protein. **(B)** Western blotting analysis for Omp2. An anti-*L. intracellularis*-positive antibody served as the primary antibody. Lane M: the prestained protein molecular weight standard; lane 1: purified Omp2; lane 2: purified GST-tagged protein.

The *Omp2* gene was correctly inserted into the pGex-6p-1 vector according to nucleotide sequencing and restriction analysis (Figure 1, lane 2). The sequence analysis of the protein was performed by DNAStar software. MegAlign analysis results showed that the homology of the amino acid sequence between GXNN strain outer membrane protein and LI0902 of PHE/MN1-00 (GenBank No. AM180252.1) is 99.1%. Compared with LI0902 of PHE/MN1-00, the 175th (Val to Alu), 223th (Glu to Gly), and 254th (Leu to Ser) amino acids generated mutation amino acids from the GXNN strain. Subsequently, the recombinant Omp2 protein was successfully expressed in BL21 (DE3) cells after it was induced by IPTG. The expressed recombinant GST-Omp2 protein was ~58.6 kDa and present in the supernatant of bacterial lysates (Figure 2A, lane 1). After purification by a GSTRAP HP column, the purity of the final products in the portion of the eluate was ~98%, as determined by image analysis of Coomassie blue-stained SDS-PAGE (Figure 2A, lane 2). Western blotting assays showed that the purified Omp2 could be specifically recognized by the anti-*L. intracellularis*-positive antibody (Figure 2B, lane 1), whereas no reactive band appeared when purified GST was used as the negative control (Figure 2B, lane 2).

### Production of Monoclonal Antibodies Against the Outer Membrane Protein of *L. intracellularis*

**Figure 3:**
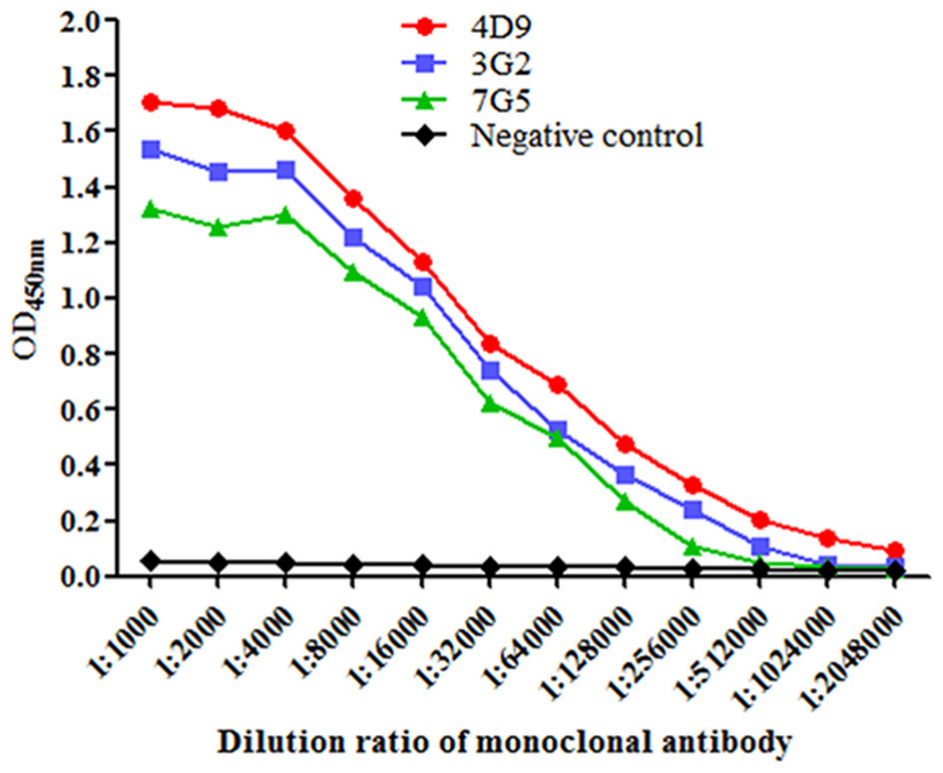
Titers of the monoclonal antibody in mouse ascites were evaluated using indirect enzyme-linked immune sorbent assay. Data were presented as mean ± SD (*n* = 3).

Purified Omp2 proteins were used to immunize BALB/c mice. To avoid obtaining mAbs against the GST tag, the GST tag of Omp2 was digested using PreScission Protease (Beyotime Biotechnology. Inc., China) before it was used as a coating antigen in the ELISA for screening (data not shown). Finally, three hybridoma cell lines named 4D9, 3G2, and 7G5 were acquired by subcloning and screening. The titers of ascitic fluids were measured by indirect ELISA, and the titers of 4D9, 3G2, and 7G5 were 1:2,048,000, 1:512,000 and 1:256,000, respectively (Figure 3).

### Subtype Identification of Monoclonal Antibodies

The results of subtype identification showed that the heavy chains of the three mAbs (4D9, 3G2, and 7G5) all belonged to IgG1, and the light chains were of the kappa type (Table 1).

**Table 1:** Subtype identification of the monoclonal antibodies.

**Subtype**	**4D9**	**3G2**	**7G5**
IgA	0.158	0.171	0.118
IgM	0.194	0.128	0.199
IgG1	2.584	2.712	2.648
IgG2a	0.171	0.184	0.149
IgG2b	0.109	0.101	0.127
IgG3	0.124	0.146	0.106
Ig-γ	0.136	0.135	0.097
Ig-k	1.673	1.713	1.748

### The Characters of the Monoclonal Antibodies

**Figure 4:**
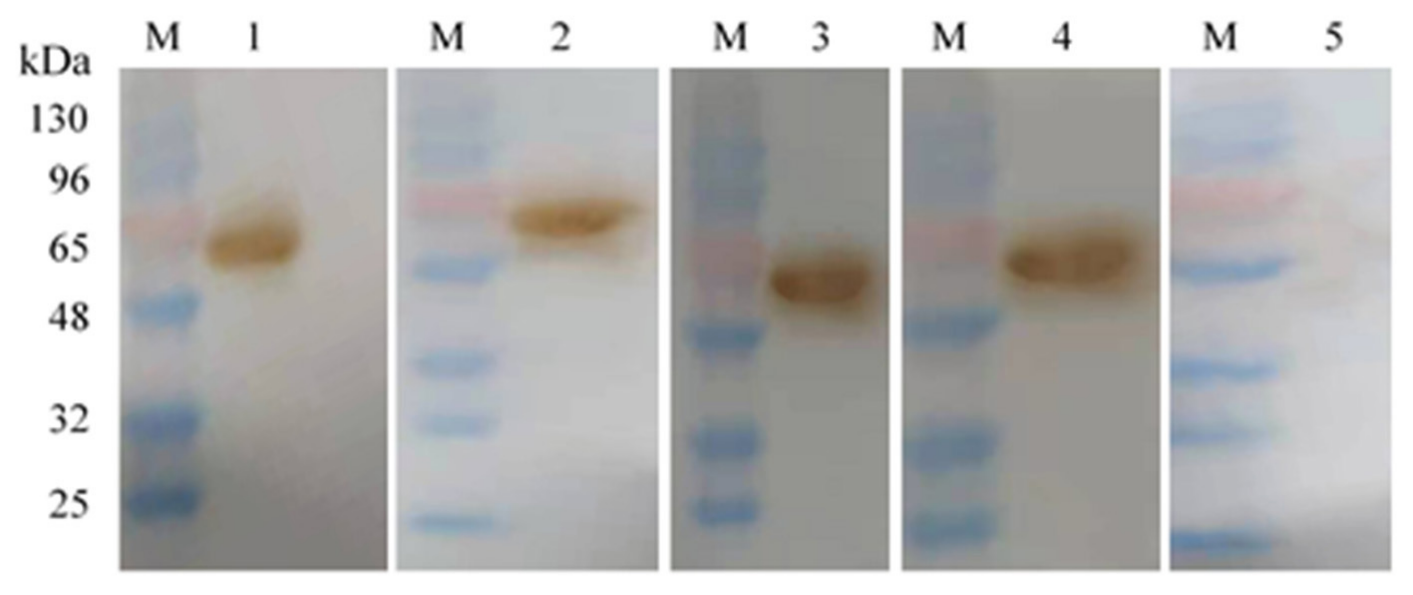
Monoclonal antibody against Omp2 of *L. intracellularis* was identified by Western blot. Lane M: the prestained protein molecular weight standard; lanes 1–3: 4D9 MAb, 3G2 MAb, and 7G5 MAb, respectively; lane 4: the mouse serum against Omp2 protein; lane 5: unimmunized normal mouse serum.

Western blotting analysis showed that all the mAbs and the positive control (the mouse serum against Omp2 protein) could react with the Omp2 protein (Figure 4, lane 1–4), whereas no reactive band appeared when the negative control (unimmunized normal mouse serum) was used (Figure 4, lane 5).

### Assessment of the Specificity of Monoclonal Antibodies

**Figure 5:**
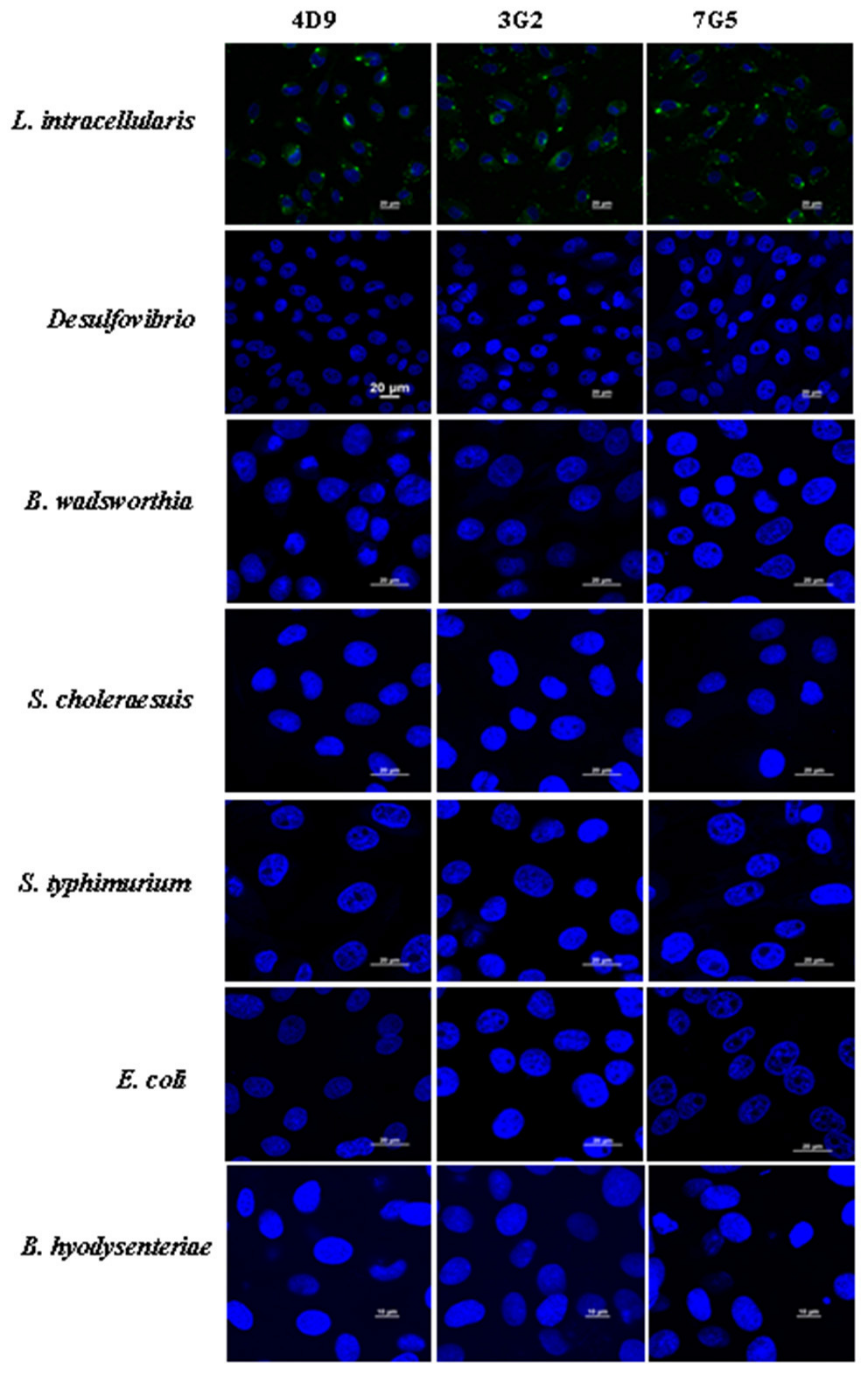
Immunofluorescence results using 4D9 MAb, 3G2 MAb, and 7G5 MAb to evaluate the cross-reactivity with *Desulfovibrio, B. wadsworthia, S. choleraesuis, S. typhimurium, E. coli*, and *B. hyodysenteriae* after incubated with IEC-18 cells for 3 h.

To test the specificity of the 4D9, 3G2, and 7G5, the IFA was conducted with pure cultures of the following bacterial strains: *Desulfovibrio, B. wadsworthia, S. choleraesuis, S. typhimurium, E. coli*, and *B. hyodysenteriae*. The results showed that 4D9, 3G2, and 7G5 did not have cross-reactions with enteric bacteria commonly found in the ileum of pigs or closely related to *L. intracellularis* (Figure 5). The results indicated that the new monoclonal antibody prepared in this study is specific against *L. intracellularis*.

### Application of Monoclonal Antibodies in Immunoflourescense Assay and Immunocytochemistry

**Figure 6:**
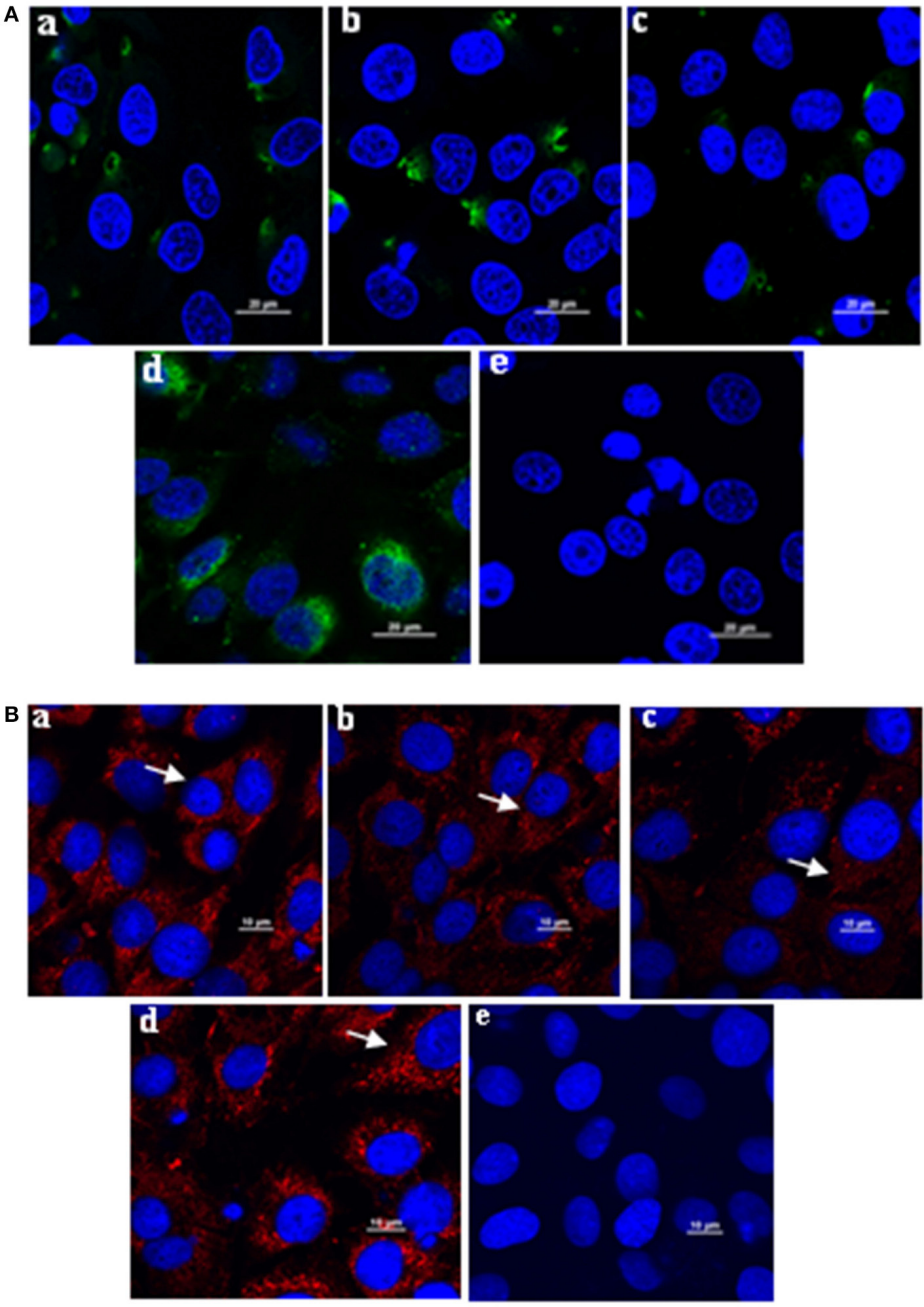
Application of the monoclonal antibody in immunofluorescence assay to monitor the *L. intracellularis* in infected monolayer cells after incubated for 3 h and 7 days, respectively. **(A)** *L. intracellularis* were incubated for 3 h; the presence of *L. intracellularis* by IFA was stained green. **(B)** *L. intracellularis* were incubated for 7 days, the presence of *L. intracellularis* was stained red. Nuclei were counterstained with DAPI (blue). The mouse serum against Omp2 protein and unimmunized normal mouse serum were used as positive and negative control. (a) 4D9 MAb; (b) 3G2 MAb; (c) 7G5 MAb; (d) positive control; (e) negative control. × 600.

**Figure 7:**
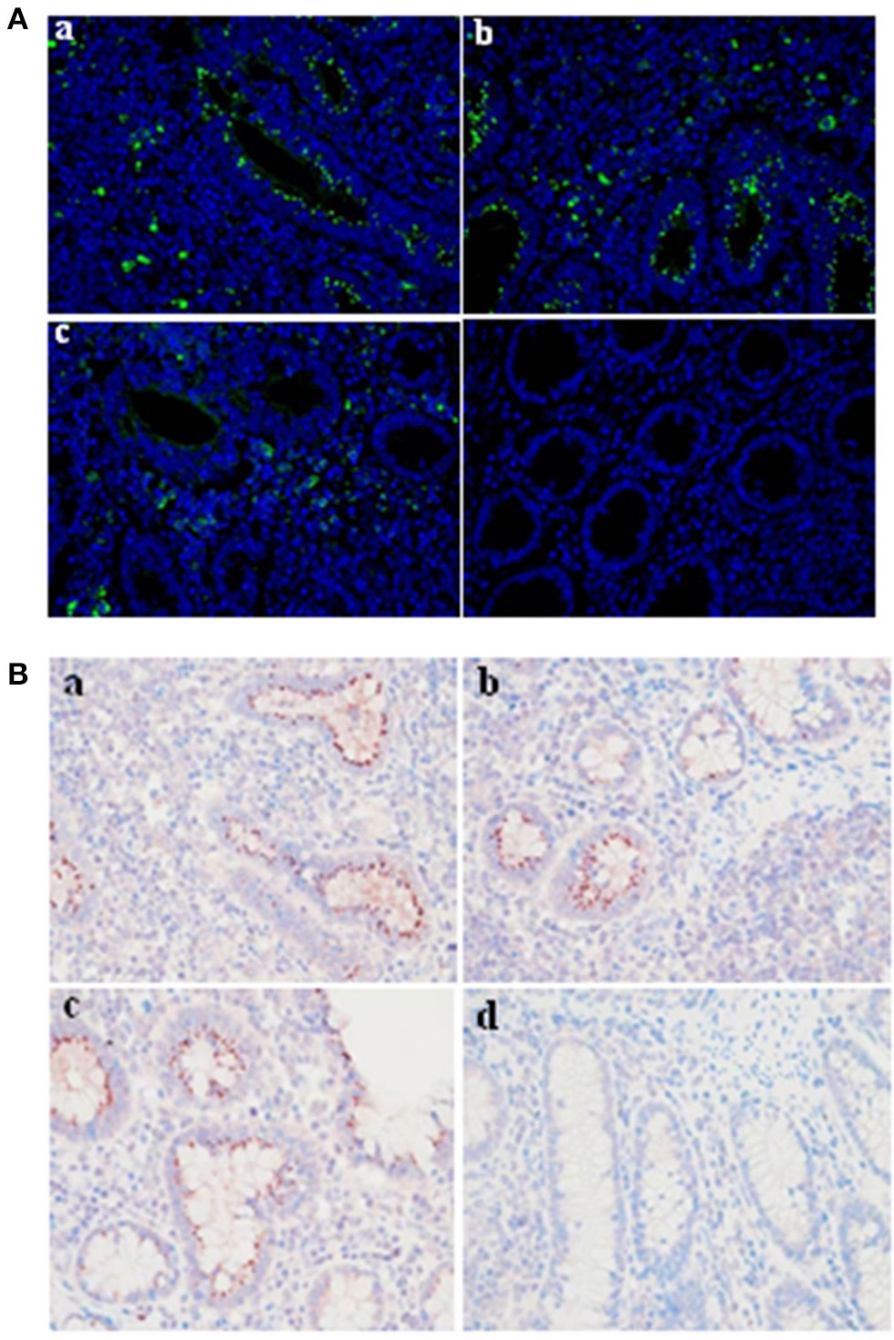
Evaluation of the monoclonal antibody by immunofluorescence and immunocytochemistry in infected tissue of PPE. IF and IHC assays were conducted in a blinded fashion on parallel sections. **(A)** Immunofluorescence assay. The presence of *L. intracellularis* by IF was detected using *L. intracellularis-*specific monoclonal antibody (4D9, 3G2, and 7G5) (green), nuclei were counterstained with DAPI (blue). **(B)** Immunocytochemistry assay. The presence of *L. intracellularis* by IHC was detected using *L. intracellularis-*specific monoclonal antibody (red), nuclei were counterstained with hematoxylin (blue). (a) 4D9 MAb; (b) 3G2 MAb; (c) 7G5 MAb; (d) negative control. × 600.

The mAbs could react with *L. intracellularis* in the infected monolayer. In particular, significant specific signals were detected using the primary antibodies against Omp2 (4D9, 3G2, and 7G5). Our findings indicated that the *L. intracellularis* in the monolayer of eukaryotic cells could be detected at 3 h post infection (Figure 6A), and highly infected cells (HIC) may be observed once the bacteria are cultivated for up to 7 days post incubation. However, no signal was detected in the negative control (Figure 6B). IFA and IHC results indicated that *L. intracellularis* antigen was visualized mainly in the crypt of the ileum from infected tissues of PPE after it reacted with *L. intracellularis*-specific monoclonal antibody, while control ileum was negative (Figures 7A,B). These results demonstrated that the new monoclonal antibody could also be applied to evaluate *L. intracellularis* antigen in infected tissues of PPE.

## Discussion

Monoclonal antibody plays an important role in the isolation and culture of *L. intracellularis*. In previous studies, the preparation of monoclonal antibodies against *L. intracellularis* mainly focused on LsaA protein and the whole bacteria protein of *L. intracellularis* (31, 32). Monoclonal antibody against surface antigens (LsaA) of *L. intracellularis* has been used as the primary antibody to detect antigen in histological tests and have been successfully detected in various field isolates from affected tissues of pigs and other animals. However, the MAb of LsaA was limited due to the lack of the *Lsa*A gene in the commercial live attenuated *L. intracellularis* vaccine. In our study, the outer membrane protein (Omp2) gene is LI0902 of *L. intracellularis*. The homology of amino acid sequence between Omp2 and LI0902 is 99.1%. Watson et al. used LC-ESI-MS/MS to analyze the two isolates of *L. intracellularis* from heavily infected epithelial cell cultures, and 19 proteins containing LI0902 were identified, and the function of Omp2 of *L. intracellularis* is thought to mediate peptidoglycan recognition and play an important role in protein–protein interactions via the vWF domain. In addition, the recombinant Omp2 protein could be recognized by sera from all but one of the infected groups and was not recognized by sera from any of the uninfected animals (33). Vannucci et al. used laser capture microdissection coupled with RNA-seq technology to characterize the transcriptional responses of infected eukaryotic cells and found that LI0902 was highly expressed in the cytoplasm of epithelial cells infected by *L. intracellularis* (34). Therefore, preparation of monoclonal antibodies against Omp2 is beneficial to research the pathogenic mechanism of *L. intracellularis* and monitor the *L. intracellularis* in infected intestinal epithelial cells.

*L. intracellularis* is an obligate intracellular bacterium that requires a specific microaerophilic environment and grows on intestinal epithelial cells (1, 35, 36). Many cell lines, such as rat small intestinal cells (IEC-18), murine fibroblast-like (McCoy), human fetal intestine (INT 407), pig kidneys (PK-15), and porcine jejunum cells (IPEC-J2), have been used to support the growth of *L. intracellularis* (18, 28). In this study, IEC-18 cells were used to culture *L. intracellularis*. To the best of our knowledge, the cells are easily contaminated by other bacteria in the intestine of pigs after inoculation with intestinal homogenate. Therefore, the isolation of *L. intracellularis* from clinical samples is extremely difficult (37). To date, there is no report regarding the successful isolation and maintenance of *L. intracellularis* infection from infected intestinal samples in China. In a previous study, *L. intracellularis* entered intestinal epithelial cells within 3 h after inoculation and was released from the vacuoles to subsequently live and proliferate in the apical cytoplasm (38). Our results also revealed that the infected cells could be precisely detected after being incubated for 3 h in a gas concentration of 8.0% O_2_, 8.8% CO_2_, and 83.2% N_2_. To obtain the high-quality intestines, it is necessary to screen the samples. Therefore, incubation for 3 h will improve the detection efficiency. Thus, in this study, to improve the detection efficiency, the infected samples were only cultured for 3 h after inoculation into IEC-18 cells.

All the three monoclonal antibodies were specific to *L. intracellularis* and did not react with pure cultures of the enteric bacteria commonly found in the ileum of pigs or closely related to *L. intracellularis*. The 4D9, 3G2, and 7G5 MAbs detected *L. intracellularis* antigen in IEC-18 cells that were positive by IFA. The different intensities of staining by IFA using different antibodies were probably because of the titer of antibodies. Furthermore, *L. intracellularis* antigen was visualized mainly in the crypt of the ileum from infected tissues of PPE after it reacted with *L. intracellularis*-specific monoclonal antibody. These results demonstrated that the new monoclonal antibody could be applied to evaluate *L. intracellularis* in infected monolayer cells and tissues of PPE. This method will make an important contribution to monitoring of the *L. intracellularis* infection.

In conclusion, the recombinant Omp2 protein of *L. intracellularis* was successfully expressed in this study through prokaryotic expression technology. Western blot results showed that the protein could react with the positive serum of *L. intracellularis*. Subsequently, the recombinant Omp2 protein was used to immunize BALB/mice, and three monoclonal antibodies against Omp2 were screened by indirect ELISA, the monoclonal antibody specific against *L. intracellularis* will be useful for the evaluation of *L. intracellularis* infection *in vivo* and *in vitro*.

## Data Availability Statement

The datasets presented in this study can be found in online repositories. The names of the repository/repositories and accession number(s) can be found in the article/supplementary material.

## Ethics Statement

The animal study was reviewed and approved by the Ethical Committee of the Faculty of Veterinary Science of Nanjing Agricultural University.

## Author Contributions

NX designed and drafted the work and wrote the manuscript. NX, JL, ML, YH, and HL performed the experiments, analyzed the data, and interpreted the results. HF helped conceive the project and edit the manuscript. All authors contributed to the article and approved the submitted version.

## Funding

This study was supported by the National Key Research and Development Program of China (2017YFD0500203), the Jiangsu Agricultural Science and Technology Innovation Fund (CX(19)2020), the Industry-University Cooperation Program of Jiangsu Province (NBG0060265-1), and the Priority Academic Program Development of Jiangsu Higher Education Institutions (PAPD).

## Conflict of Interest

The authors declare that the research was conducted in the absence of any commercial or financial relationships that could be construed as a potential conflict of interest.

## Publisher's Note

All claims expressed in this article are solely those of the authors and do not necessarily represent those of their affiliated organizations, or those of the publisher, the editors and the reviewers. Any product that may be evaluated in this article, or claim that may be made by its manufacturer, is not guaranteed or endorsed by the publisher.
